# The Differential Effects of Social Media on Depressive Symptoms and Suicidal Ideation Among the Younger and Older Adult Population in Hong Kong During the COVID-19 Pandemic: Population-Based Cross-sectional Survey Study

**DOI:** 10.2196/24623

**Published:** 2021-05-25

**Authors:** Xue Yang, Benjamin H K Yip, Arthur D P Mak, Dexing Zhang, Eric K P Lee, Samuel Y S Wong

**Affiliations:** 1 Jockey Club School of Public Health and Primary Care Faculty of Medicine The Chinese University of Hong Kong Hong Kong Hong Kong; 2 The Chinese University of Hong Kong Shenzhen Research Institute Shenzhen China; 3 Department of Psychiatry The Chinese University of Hong Kong Hong Kong Hong Kong

**Keywords:** social media, depression, suicidal ideation, social loneliness, posttraumatic stress, suicide, mental health, COVID-19, loneliness, age, mediation

## Abstract

**Background:**

Social media has become a ubiquitous part of daily life during the COVID-19 pandemic isolation. However, the role of social media use in depression and suicidal ideation of the general public remains unclear. Related empirical studies were limited and reported inconsistent findings. Little is known about the potential underlying mechanisms that may illustrate the relationship between social media use and depression and suicidal ideation during the COVID-19 pandemic.

**Objective:**

This study tested the mediation effects of social loneliness and posttraumatic stress disorder (PTSD) symptoms on the relationship between social media use and depressive symptoms and suicidal ideation, as well as the moderation effect of age on the mediation models.

**Methods:**

We administered a population-based random telephone survey in May and June 2020, when infection control measures were being vigorously implemented in Hong Kong. A total of 1070 adults (658 social media users and 412 nonusers) completed the survey. Structural equation modeling (SEM) and multigroup SEM were conducted to test the mediation and moderation effects.

**Results:**

The weighted prevalence of probable depression was 11.6%; 1.6% had suicidal ideation in the past 2 weeks. Both moderated mediation models of depressive symptoms (*χ*^2^_62_=335.3; *P*<.05; comparative fit index [CFI]=0.94; nonnormed fit index [NNFI]=0.92; root mean square error of approximation [RMSEA]=0.06) and suicidal ideation (*χ*^2^_34_=50.8; *P*<.05; CFI=0.99; NNFI=0.99; RMSEA=0.02) showed acceptable model fit. There was a significantly negative direct effect of social media use on depressive symptoms among older people (β=–.07; *P*=.04) but not among younger people (β=.04; *P*=.55). The indirect effect via PTSD symptoms was significantly positive among both younger people (β=.09; *P*=.02) and older people (β=.10; *P*=.01). The indirect effect via social loneliness was significant among older people (β=–.01; *P*=.04) but not among younger people (β=.01; *P*=.31). The direct effect of social media use on suicidal ideation was not statistically significant in either age group (*P*>.05). The indirect effects via PTSD symptoms were statistically significant among younger people (β=.02; *P*=.04) and older people (β=.03; *P*=.01). Social loneliness was not a significant mediator between social media use and suicidal ideation among either age group (*P*>.05).

**Conclusions:**

Social media may be a “double-edged sword” for psychosocial well-being during the COVID-19 pandemic, and its roles vary across age groups. The mediators identified in this study can be addressed by psychological interventions to prevent severe mental health problems during and after the COVID-19 pandemic.

## Introduction

Unprecedented control measures, such as lockdown, quarantine, social distancing, and home confinement, have been implemented to contain the spread of COVID-19, an infectious disease caused by a coronavirus that was newly discovered in 2019 [1,2]. These measures have brought marked changes in our social worlds and digital lifestyles within a short time frame. As a large proportion of the global population hunkers down in isolation away from their family and friends, social media and social networking sites (SNSs) have become a crutch for human connection and information sharing [3]. Although the World Health Organization (WHO) has expressed support for the gaming industry’s online social media campaign #PlayApartTogether, which incorporates WHO guidelines on coronavirus prevention [4], the role of social media use in the mental health of the general public during the COVID-19 pandemic remains unclear. Related empirical studies have been limited and have reported inconsistent findings [5-8]. The inconsistent results highlight the need to explore the potential underlying mechanisms that may illustrate the link between social media use and mental health during the COVID-19 pandemic; however, we did not identify such studies. In addition, there have been growing concerns about people experiencing increased suicidal ideation or self-harming behavior during the COVID-19 pandemic isolation [9]. No study tested whether or how social media use status may influence suicidal ideation during the COVID-19 pandemic.

Reduced social loneliness and increased trauma-related stress (eg, posttraumatic stress disorder [PTSD] symptoms) may serve as two important psychosocial mechanisms that explain the relationship between social media use and depression and suicidal ideation. Social loneliness refers to the situation where an individual has a smaller number of relationships, stemming from the absence of a broader group of contacts or an engaging social network, such as friends, colleagues, and neighbors. Meanwhile, social media has been demonstrated to play an important role in forming and maintaining social networks and social capital, which may counter social loneliness [10-12]. While isolation during the COVID-19 pandemic may have reduced physical interpersonal encounters and increased social loneliness, social media can maintain social connections (eg, Shah et al [13]). Thus, social media may help to reinforce interpersonal resources and ameliorate social loneliness, which may help people cope with stress and mitigate the adverse impact of the COVID-19 pandemic on mental health [14,15].

On the other hand, intensified trauma-related stress and PTSD symptoms may explain the positive association between social media use and mental health (eg, Chao et al [5] and Riehm et al [6]). Social media may allow for the spread of rumors, fake news, and negative emotions, such as hopelessness, anxiety, and fear (eg, Depoux et al [16] and Kramer et al [17]). Ubiquitous and repeated social media exposure to anxiety-provoking topics related to the health crisis can also lead users to inaccurately estimate the threat of infection within their communities [18]. This can trigger acute and posttraumatic stress responses as well as panic responses during collective traumatic events, which may, in turn, aggravate depressive symptoms and suicidal ideation during a pandemic [19].

Theoretically, the proposed mediation effects of social loneliness and PTSD symptoms can be supported by the conservation of resources (COR) theory [20]. This theory suggests that perceived and actual loss or gain of interpersonal resources (eg, social connection and loneliness) and personal resources (eg, perceived stress and self-efficacy) serves as the central mechanism that explains how people may develop psychological distress. This theory has been applied to explain the relationships between the use of digital technology and psychological well-being; that is, social media can significantly affect the psychosocial resources of users, which, in turn, affect their mental health and distress (eg, Feldman et al [21] and van der Velden et al [22]). Social loneliness and perceived trauma-related stress are well-documented interpersonal and personal factors of severe mental health problems [23,24]. However, we have not identified any studies that tested their mediation effects on the relationship between social media use and mental health during collective traumatic events.

Furthermore, an increasing number of older adults have been using the internet and social media. In Hong Kong, people aged 45 years or above have caught up rapidly with their social media participation rate (ie, 78% in 2018) [25]. Social media applications are considered helpful in reducing loneliness and enhancing well-being among older adults, while concerns about the negative consequences on well-being have been highlighted in some studies (eg, Leist [26] and Berryman et al [27]). Most research on the effects of social media use has focused on younger people only, with few studies conducted among older people (eg, van der Velden et al [22]). It is unclear how age may moderate the effect of social media use on psychosocial status during the COVID-19 pandemic.

In this study, we randomly recruited both social media users and nonusers to create a representative sample of the Hong Kong population; we examined whether and how social media use is associated with depressive symptoms and suicidal ideation through two psychosocial processes: social loneliness and PTSD symptoms. We hypothesized the following:

1. Social media use would be negatively associated with social loneliness; in turn, loneliness would be positively associated with depressive symptoms and suicidal ideation.
2. Social media use would be positively associated with PTSD symptoms; in turn, PTSD symptoms would be positively associated with depressive symptoms and suicidal ideation.

In addition, we also tested whether these mediation effects would be constant among younger and older people.

## Methods

### Recruitment of Participants

We administered a population-based, random telephone survey between May 14 and June 4, 2020, when infection control measures (eg, social distancing, business restrictions, and border control) were being vigorously implemented in Hong Kong. Participant inclusion criteria included the following: (1) Chinese speaking, (2) 18 years old or above, and (3) Hong Kong resident (ie, holder of a Hong Kong identification card). The telephone interviews were conducted between 6 PM and 10 PM in order to avoid undersampling working individuals. The interviewers were well trained and had at least 6 months of interviewing experience. They were supervised on site by a senior project coordinator. Telephone numbers were randomly drawn from the latest residential telephone directory by a random phone number generator program. Telephone numbers were selected randomly from an updated landline telephone directory as seed numbers. Another three sets of numbers were then generated using the randomization of the last two digits to recruit unlisted numbers. Eligible household members whose day and month of birth was closest to the survey date were invited to join the study. Two follow-up calls were conducted for unanswered calls before a telephone number was considered invalid. Verbal informed consent was obtained from the participants. The anonymous interview took 10 to 15 minutes. No incentive was given to the participants. Of the 1882 eligible participants identified and invited, 1070 completed the interviews, resulting in a modest response rate (56.9%).

### Ethical Approval

The study was approved by the Survey and Behavioural Research Ethics Committee of the corresponding author’s affiliated university, the Chinese University of Hong Kong (reference No. SBRE-19-645). The study followed the ethical standards of the responsible committee on human experimentation, institutional and national, and of the Helsinki Declaration of 1975, as revised in 2000.

### Measures

#### Status of Social Media Use

The participants were asked whether they had used an SNS in the past 12 months, such as Facebook, Twitter, WhatsApp, or WeChat, which are platforms for communicating with one another [28]. Those who said “yes” to the question were further asked how many hours per day they had spent, on average, on these SNSs during the COVID-19 pandemic. Similar questions were used in previous studies on social media use [8,29,30].

#### PTSD Symptoms

The 8-item Posttraumatic Stress Disorder scale (PTSD-8) [31] was used to assess posttraumatic stress responses and symptoms in the past month. The items correspond to the DSM-IV (Diagnostic and Statistical Manual of Mental Disorders, Fourth Edition) criteria for PTSD. They are answered on a 4-point Likert scale, ranging from 1 (not at all) to 4 (all the time). Higher summed scores indicate greater symptoms of PTSD. The internal consistency as measured by Cronbach α was .76 and was acceptable in the current sample.

#### Social Loneliness

The 3-item social loneliness subscale of the De Jong Gierveld Loneliness Scale [32] was used to assess social loneliness during the COVID-19 pandemic. Response options include *no*, *more or less*, and *yes*. Summed scores range from 3 to 9. Higher scores suggest higher levels of loneliness. The Cronbach α for this scale was .94.

#### Depressive Symptoms

The 10-item Center for Epidemiologic Studies Depression Scale (CESD-10) assessed depressive symptoms during the past week [33]. This is a short version of the CESD-20 and has good reliability and validity [33]. A cutoff point of 10 or higher denotes probable depression; this was predictive of a depression diagnosis [34,35]. Items are rated on a 4-point Likert scale, ranging from 0 (less than 1 day) to 3 (5 to 7 days). The Chinese version of the scale was validated in the Hong Kong population [36]. The Cronbach α was .78 in the current sample.

#### Suicidal Ideation

Item 9 of the 9-item Patient Health Questionnaire (PHQ-9) [37] (ie, “How often have you been bothered by the following problem: Thoughts that you would be better off dead, or thoughts of hurting yourself in some way?”) was used to assess the frequency of suicidal ideation in the past 2 weeks. Participants rated the question on a 4-point Likert scale, ranging from 0 (not at all) to 3 (almost every day). A score of 0 indicates having no suicidal ideation, while a score of 1 or higher indicates having suicidal ideation in the past 2 weeks. The Chinese version has been used in previous studies [38].

The participants were also asked to report their sociodemographic information, including sex, age, current marital status, educational level, income, health status, and mandatory quarantine status (ie, whether one had been subjected to compulsory quarantine at designated places—home, hotel, or other accommodation—under government order for COVID-19 infection control).

### Statistical Analyses

Descriptive statistics were computed for both background and psychological variables. Age-standardized weighted prevalence of probable depression was calculated by the direct method and the age distribution for the 2020 census population. Simple logistic regression analyses were conducted to test the associations between background, independent, and mediation variables and probable depression and suicidal ideation. Odds ratios and 95% CIs were reported. Structural equation modeling (SEM) was conducted to test the proposed mediation models of depressive symptoms and suicidal ideation. For the variables of PTSD symptoms and depressive symptoms, indicators were created by the item parceling method. Since the two scales are unidimensional, the random method of combining items was used to create item parcels. For the latent factors of social loneliness, all three individual items of the scale were used as indicators. The observed variable of suicidal ideation was created by using item 9 of the PHQ-9. Goodness of fit was tested by using the chi-square test, the comparative fit index (CFI), the nonnormed fit index (NNFI), and the root mean square error of approximation (RMSEA). Standardized regression coefficients (β) and 95% CIs were reported. Bootstrapping based on 5000 bootstrap samples was performed to test for indirect effects. A statistically significant indirect effect would be observed when the CI did not include zero. Multigroup SEM analyses were conducted to test the moderation effect of age on the mediation models. The age of retirement of most people in Hong Kong ranges from 55 to 65 years [39]. Hence, participants aged 18 to 55 years were classified as younger adults and those older than 55 years were classified as older adults. The level of statistical significance was .05. SPSS, version 21.0, and Amos (IBM Corp) were used to conduct statistical analysis.

## Results

The background characteristics of the participants are presented in Table 1. The sample of 1070 participants included 367 (34.3%) young adults and 684 (63.9%) older adults; 60.4% (646/1070) of the participants reported that they used social media in the past year and were classified as social media users. The weighted prevalence of probable depression was 11.6%. Younger adults (14.8%) had higher weighted prevalence of probable depression than older adults (8.4%). A total of 1.6% (17/1070) of the participants had suicidal ideation in the past 2 weeks (younger adults: 10/367, 2.7%; older adults: 7/684, 1.0%).

Associations between the background or mediator variables and probable depression and suicidal ideation are presented in Table 2. The significant background variables of probable depression included age, current marital status, educational level, monthly household income, and mental health status before and during the COVID-19 pandemic. Social media use status, time spent on social media, PTSD symptoms, and social loneliness were significantly and positively associated with depressive symptoms. Income, being diagnosed with mental health problems before or during the COVID-19 pandemic, mandatory quarantine status, and PTSD symptoms were positively associated with suicidal ideation.

**Table 1:** Background characteristics of the participants recruited from the adult population in Hong Kong during the COVID-19 pandemic.

Background characteristic		All participants (N=1070)	Younger adults (aged 18-55 years; n=367)	Older adults (older than 55 years; n=684)	*P* value^a^	
**Sex, n (%)**						.01
	Male	346 (32.3)	137 (37.3)	204 (29.8)		
	Female	724 (67.7)	230 (62.7)	480 (70.2)		
**Age group (years), n (%)**						N/A^b^
	18-35	115 (10.7)	N/A	N/A		
	36-55	252 (23.6)	N/A	N/A		
	56-65	301 (28.1)	N/A	N/A		
	>65	383 (35.8)	N/A	N/A		
	Refused to answer	19 (1.8)	N/A	N/A		
**Current marital status, n (%)**						<.001
	Single	201 (18.8)	148 (40.3)	50 (7.3)		
	Cohabiting or married	745 (69.6)	209 (56.9)	528 (77.2)		
	Separated, divorced, or widowed	107 (10.0)	5 (1.4)	101 (14.8)		
	Refused to answer or missing value	17 (1.6)	5 (1.4)	5 (0.7)		
**Educational level, n (%)**						<.001
	Primary school or below	355 (33.2)	7 (1.9)	345 (50.4)		
	Secondary school	384 (35.9)	139 (37.9)	239 (34.9)		
	College or above	294 (27.5)	211 (57.5)	81 (11.8)		
	Refused to answer	37 (3.5)	10 (2.7)	19 (2.8)		
**Monthly household income (HK $^c^), n (%)**						<.001
	≤20,000	627 (58.6)	92 (25.1)	526 (76.9)		
	20,001-30,000	124 (11.6)	77 (21.0)	47 (6.9)		
	30,001-50,000	103 (9.6)	64 (17.4)	39 (5.7)		
	>50,000	84 (7.9)	62 (16.9)	22 (3.2)		
	Refused to answer or missing value	132 (12.3)	72 (19.6)	50 (7.3)		
**Have chronic diseases^d^, n (%)**						<.001
	No	716 (66.9)	334 (91.0)	369 (53.9)		
	Yes	354 (33.1)	33 (9.0)	315 (46.1)		
**Diagnosed with mental health problems^e^ before the pandemic, n (%)**						.25
	No	1045 (97.7)	361 (98.4)	665 (97.2)		
	Yes	25 (2.3)	6 (1.6)	19 (2.8)		
**Diagnosed with mental health problems^e^ during the pandemic, n (%)**						.64
	No	1050 (98.1)	361 (98.4)	670 (98.0)		
	Yes	20 (1.9)	6 (1.6)	14 (2.0)		
**Subjected to mandatory quarantine** **, n (%)**						.02
	No	1055 (98.6)	358 (97.5)	679 (99.3)		
	Yes	15 (1.4)	9 (2.5)	5 (0.7)		
**Social media user in the past year, n (%)**						<.001
	No	412 (38.5)	25 (6.8)	379 (55.4)		
	Yes	658 (61.5)	342 (93.2)	305 (44.6)		
**Hours spent per day on social media during the pandemic (n=658)** **, n (%)**						<.001
	0.0-2.0	232 (35.3)	89 (26.0)	137 (44.9)		
	2.5-4.0	261 (39.7)	133 (38.9)	126 (41.3)		
	4.5-6.0	110 (16.7)	77 (22.5)	30 (9.8)		
	>6.0	55 (8.4)	43 (12.6)	12 (3.9)		
Posttraumatic stress disorder symptoms, mean (SD)^f^		4.2 (3.8)	4.7 (3.6)	4.0 (3.9)	.008	
Social loneliness, mean (SD)^g^		4.4 (1.7)	4.4 (1.8)	4.3 (1.7)	.76	

^a^Based on chi-square tests or independent-samples *t* tests where appropriate.

^b^N/A: not applicable; the number of participants in each age group was reported for the total sample only (hence, the *P* value was not calculated).

^c^A currency exchange rate of HK $1=US $0.1287 is applicable.

^d^Chronic diseases included hypertension, diabetes, cancer, etc.

^e^Mental health problems included depression, anxiety, insomnia, etc.

^f^The 8-item Posttraumatic Stress Disorder scale (PTSD-8) was used to assess posttraumatic stress responses and symptoms in the past month. Summed scores range from 0 to 23; higher summed scores indicate greater symptoms of PTSD.

^g^The 3-item social loneliness subscale of the De Jong Gierveld Loneliness Scale was used to assess social loneliness. Summed scores range from 3 to 9; higher scores suggest higher levels of loneliness.

**Table 2:** Associations between the background or mediator variables and depressive symptoms and suicidal ideation among the adult population in Hong Kong during the COVID-19 pandemic (N=1070).

Variable		Probable depression OR^a^ (95% CI)	*P* value	Suicidal ideation OR (95% CI)	*P* value
**Sex**					
	Male	1^b^		1	
	Female	1.09 (0.71-1.67)	.69	0.68 (0.26-1.80)	.44
**Age (years)**					
	18-35	1		1	
	36-55	0.90 (0.49-1.66)	.73	0.68 (0.19-2.45)	.55
	56-65	0.55 (0.29-1.04)	.07	0.37 (0.09-1.52)	.17
	>65	0.39 (0.21-0.75)	.004	0.22 (0.05-0.99)	.049
**Current marital status (n=1053)**					
	Single	1		1	
	Cohabiting or married	0.61 (0.39-0.96)	.03	0.53 (0.18-1.58)	.26
	Separated, divorced, or widowed	0.46 (0.20-1.04)	.06	0.75 (0.14-3.91)	.73
**Educational level (n=1033)**					
	Primary school or below	1		1	
	Secondary school	1.41 (0.83-2.39)	.21	2.18 (0.56-8.49)	.26
	College or above	2.45 (1.46-4.09)	.001	2.86 (0.73-11.17)	.13
**Monthly household income (HK $^c^; n=938)**					
	≤20,000	1		1	
	20,001-30,000	2.41 (1.38-4.18)	.002	1.45 (0.30-7.07)	.64
	30,001-50,000	1.56 (0.80-3.04)	.20	2.66 (0.68-10.45)	.16
	>50,000	2.78 (1.50-5.15)	.001	4.43(1.27-15.46)	.02
**Have chronic diseases^d^**					
	No	1		1	
	Yes	0.84 (0.55-1.29)	.43	0.84 (0.29-2.41)	.75
**Diagnosed with mental health problems^e^ before the pandemic**					
	No	1		1	
	Yes	12.44 (5.50-28.15)	<.001	15.12 (4.55-50.26)	<.001
**Diagnosed with mental health problems^e^ during the pandemic**					
	No	1		1	
	Yes	40.21 (13.18-122.72)	<.001	28.83 (9.03-92.08)	<.001
**Subjected to mandatory quarantine**					
	No	1		1	
	Yes	2.19 (0.61-7.89)	.23	10.67 (2.21-51.45)	.003
**Social media use in the past year**					
	No	1		1	
	Yes	1.98 (1.27-3.10)	.003	2.96 (0.85-10.38)	.09
**Hours spent per day on social media during the pandemic (n=658)**					
	0.0-2.0	1		1	
	2.5-4.0	1.29 (0.72-2.29)	.39	0.29 (0.08-1.08)	.06
	4.5-6.0	2.12 (1.10-4.08)	.02	0.23 (0.03-1.82)	.16
	>6.0	2.12 (0.94-4.79)	.07	0.46 (0.06-3.70)	.46
Posttraumatic stress disorder symptoms		1.38 (1.31-1.46)	<.001	1.21 (1.11-1.31)	<.001
Social loneliness		1.17 (1.05-1.30)	.004	1.20 (0.93-1.54)	.16

^a^OR: odds ratio; based on logistic regression analyses.

^b^Variable items with a value of 1 are the reference items.

^c^A currency exchange rate of HK $1=US $0.1287 is applicable.

^d^Chronic diseases included hypertension, diabetes, cancer, etc.

^e^Mental health problems included depression, anxiety, insomnia, etc.

**Figure 1:**
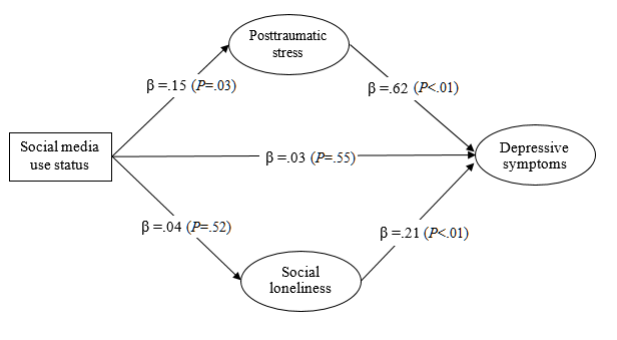
The proposed mediation model of depressive symptoms with standardized regression coefficients (β) among younger adults in Hong Kong during the COVID-19 pandemic (n=367).

**Figure 2:**
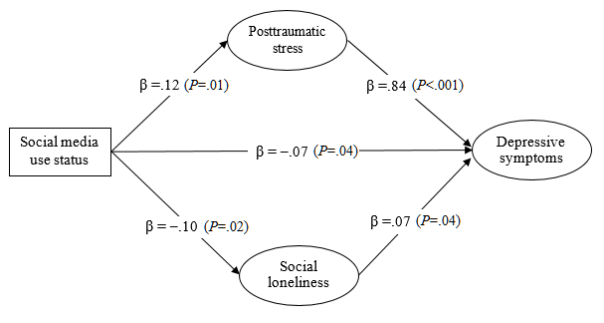
The proposed mediation model of depressive symptoms with standardized regression coefficients (β) among older adults in Hong Kong during the COVID-19 pandemic (n=684).

Both the measurement model (*χ*^2^_30_=294.3; *P*<.05; CFI=0.95; NNFI=0.92; RMSEA=0.08) and the structural model of depressive symptoms (**χ**^2^_31_=294.6; *P*<.05; CFI=0.95; NNFI=0.92; RMSEA=0.08) showed acceptable model fit. Multigroup SEM analyses further revealed that the mediation model fitted the data well across younger and older adults (*χ*^2^_62_=335.3; *P*<.05; CFI=0.94; NNFI=0.92; RMSEA=0.06). As Figures 1 and 2 show, there was a significantly negative direct effect of social media use on depressive symptoms among older people (β=–.07; *P*=.04) (Figure 2) but not among younger people (β=.03; *P*=.55) (Figure 1). The indirect effect via PTSD symptoms was significantly positive among both younger people (β=.09, 95% CI .02-.14; *P*=.02) (Figure 1) and older people (β=.10, 95% CI .05-.16; *P*=.01) (Figure 2). The indirect effect via social loneliness was significant among older people (β=–.01, 95% CI –.02 to –.001; *P*=.04) (Figure 2) but not among younger people (β=.01, 95% CI –.01 to .03; *P*=.31) (Figure 1).

**Figure 3:**
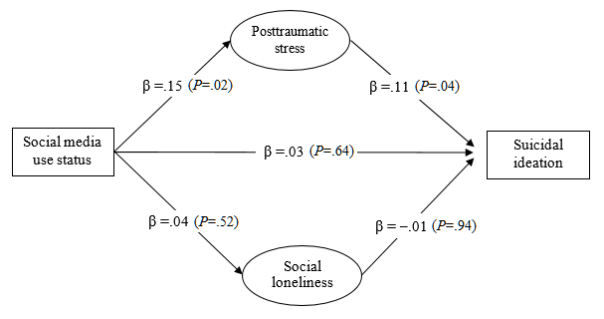
The proposed mediation model of suicidal ideation with standardized regression coefficients (β) among younger adults in Hong Kong during the COVID-19 pandemic (n=367).

**Figure 4:**
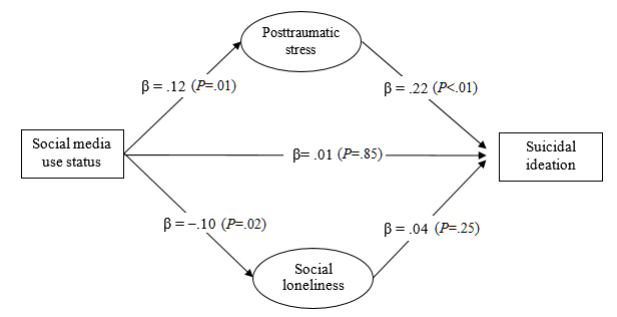
The proposed mediation model of suicidal ideation with standardized regression coefficients (β) among older adults in Hong Kong during the COVID-19 pandemic (n=684).

Both the measurement model (*χ*^2^_16_=34.4; *P*<.05; CFI=0.99; NNFI=0.99; RMSEA=0.03) and the structural model of suicidal ideation (*χ*^2^_17_=34.7; *P*<.05; CFI=0.99; NNFI=0.99; RMSEA=0.03) showed excellent model fit. Multigroup SEM analyses showed that the mediation model fitted the data well across age groups (*χ*^2^_34_=50.8; *P*<.05; CFI=0.99; NNFI=0.99; RMSEA=0.02). As Figures 3 and 4 show, the direct effect of social media use on suicidal ideation was not statistically significant in either age group (*P*>.05). The indirect effects via PTSD symptoms were statistically significant among younger people (β=.02, 95% CI .001-.06; *P*=.04) (Figure 3) and older people (β=.03, 95% CI .01-.06; *P*=.01) (Figure 4). Social loneliness was not a significant mediator between social media use and suicidal ideation among younger and older adults.

## Discussion

### Principal Findings

This population-based study investigated the prevalence of probable depression and suicidal ideation in Hong Kong adults during the COVID-19 pandemic. Furthermore, the study tested a complex relationship between social media use and mental health (ie, depressive symptoms and suicidal ideation). Findings suggest that this relationship could be explained by the mediation effects of PTSD symptoms and social loneliness and moderated by age. Specifically, social media use was indirectly and positively associated with depressive symptoms and suicidal ideation through PTSD symptoms in both younger and older adults. In addition, social media use was directly and indirectly associated with depressive symptoms through social loneliness in older adults.

The weighted prevalence of probable depression was higher than that reported in pre–COVID-19 research conducted in 2007 (11.6% versus 8.6%) [40]. It may suggest an increase in mental distress among the Hong Kong population during the COVID-19 pandemic. Some subgroups may need particular attention, as they showed higher risks of depression or suicidal ideation, including those who were younger or single, had higher educational levels, had higher household incomes, had been diagnosed with mental health problems before or during the COVID-19 pandemic, had been under mandatory quarantine, used social media, or spent more hours on social media during the COVID-19 pandemic. Higher scores of depression among the younger sample seem to confirm findings from both COVID-19 and pre–COVID-19 studies [41,42]: the younger participants tended to obtain a large amount of information from social media that could easily trigger stress. Interestingly, the results appear to suggest that people with higher social capital, such as having higher educational levels, having higher household incomes, or using social media, may be more vulnerable to mental distress during the COVID-19 pandemic. Recent studies reported similar findings and suggested that it may be because these groups tend to have higher self-awareness of, and concern about, their health [41,43]. Time spent on social media was positively associated with depressive symptoms during the COVID-19 pandemic, consistent with recently published studies [5-7]. It may be due to the fact that during the COVID-19 pandemic, spending excessive time on social media implies more social media exposure to COVID-19 news and greater likelihood of experiencing the infodemic and emotional contagion through online social networks [44]. These findings are susceptible to reverse causality, whereby mental distress might lead to rumination on social media.

Furthermore, this study brings novel information to the field about the underlying mechanisms of the relationships between social media use and depression and suicidal ideation. The proposed mediation models based on the COR theory were well supported by the acceptable model fit. This is the first study that applied this theory to understand the roles of social media use in the context of the COVID-19 pandemic. From the COR perspectives, people strive to develop, maintain, or restore important resources, such as social relationships, well-being, and a low state of stress, and a loss of these resources can, in turn, lead to mental health problems [20,45]. Our SEM results suggest that during the COVID-19 crisis, social media use might intensify PTSD symptoms, which were, in turn, associated with more depressive symptoms and suicidal ideation; these mediation effects are broadly applicable to both younger and older adults. Our findings support the assertion that the heightened PTSD symptoms due to media exposure to collective crises may have profound repercussions for mental health [46]. Consistently, recent studies also suggested that social media use, especially long times spent on social media for COVID-19–related information, was positively associated with a range of negative psychological statuses, including negative affect, mental distress, anxiety, and depression [5-7]. Previous studies in the contexts of other infectious diseases and traumatic events (eg, the Ebola virus disease outbreak) also argued that a state of stress could be triggered and intensified by social media exposure [16,17,47]. Reverse causality may also be applicable, as people experiencing more stress and mental health problems may tend to use social media to escape from the real world, which is stressful during the COVID-19 pandemic. Follow-up studies are warranted to better understand their dynamic relationships at different stages of the COVID-19 pandemic and to monitor whether social media exposure during the crisis would lead to PTSD in the long run. Other personal resources, such as information deficiency as well as positive and negative affect, may also serve as mediators between social media use and mental health. In addition, other mental and emotional statuses, such as anxiety, can be induced by the false, fearful, and anxiety-increasing messages—due to politicization, rumination, sensationalizing, or catastrophizing—spread by social media, and can explain the development of depression and suicidal ideation [48]. Pervasive uncertainty and hopelessness increased by repeated exposures to online information related to the health crisis may also be a robust predictor of suicidal ideation and suicide, especially for vulnerable groups (eg, people who need ongoing mental health care) [49]. These potential mediators should be explored in future work.

On the other hand, social media use might indirectly reduce depression because it can provide opportunities to maintain and enhance interpersonal resources (eg, reduced social loneliness) by using SNSs, such as Facebook, WeChat, and WhatsApp, during the COVID-19 pandemic isolation. This result provides preliminary empirical evidence for the assertion of recently published commentaries [50]. However, such mediation effects might vary across age groups. We found that social media use might only benefit older adults’ mental health by ameliorating their social loneliness, as the negative direct and indirect effects of social media use on depressive symptoms through social loneliness were statistically significant among older adults but not among younger adults. Consistently, a study by Cotten et al, which was conducted among retired residents, estimated that internet use reduced depression (CESD-8 score ≥4) by about 30% among this older group [51]; in addition, van Ingen et al found that social media use was predictive of social loneliness among older adults [52]. The age differences may be due to the fact that for younger people, social media is the “real” and default mode of social networks, which is less likely to change because of the COVID-19 pandemic. However, older adults might have taken up social media because of the COVID-19 pandemic—or the 2019-2020 social movement in Hong Kong—and that would have a more dynamic implication for older adults than for younger adults. The life span theory of selective optimization with compensation [53] can also be used to explain such age differences. This theory suggests that older people may experience various age-related losses, including those in social reserves [54]. Thus, social media use may be a particularly useful strategy that older adults can use to compensate for reduced mobility and social connection and that can contribute to their own successful aging and well-being [55]. Social media use may help to gain other interpersonal and social resources, such as social support, timely health communication, and access to and utilization of technology-based health care services, which may explain the relationship between social media use and mental health.

Unexpectedly, social loneliness was not a significant mediator between social media use and suicidal ideation because it was not significantly associated with suicidal ideation. Inconsistently, previous studies found that loneliness and social connection were significant interpersonal factors of suicidal ideation [23,24]. The insignificant association in our study may be because the lack of social networks during the COVID-19 pandemic isolation has been seen as normal, which may temporally mitigate its harmful effect on hopelessness and suicidal ideation. However, given that social connection is a basic human psychological need [56], the long-term effects of social loneliness on suicidal ideation should be investigated.

### Implications

The positive mediation effect of PTSD symptoms among younger and older people and the negative mediation effect of social loneliness among older people suggest that social media use may have both beneficial and harmful effects on mental health during the COVID-19 pandemic, and that age plays a significant role. Notably, the mediation effect of PTSD symptoms was larger than that of social loneliness. This is consistent with the principle of the COR theory, in that resource loss is disproportionately more salient than resource gain [20]. The effect sizes of both mediation effects were relatively small. Hence, the results should be interpreted with caution. Nevertheless, these findings highlight the importance of further exploration of underlying mechanisms in understanding the complex relationship between social media use and mental health during different times across the life span and in different social contexts.

Such results have important practical and political implications. First, the high prevalence of mental health problems during the COVID-19 pandemic is a significant public health concern, and high-risk groups (eg, younger people) need particular attention from health care service providers. From a public health perspective, there are effective mental health interventions (eg, cognitive behavioral therapy and mindfulness-based interventions) available, which can be delivered via the internet during the COVID-19 pandemic isolation. Second, since the COVID-19 pandemic might persist and a digital lifestyle could become inevitable, it is important to understand the psychological mechanisms that may explain how digital technology users and nonusers may be different in psychosocial status and mental health. Our studied psychosocial mediators can be modified by interventions and can be used to guide prevention programs for mental health problems. For example, health education programs and public health strategies are recommended to enhance awareness of digital literacy, strategic social media use, and potential harms of social media use in the general public to reduce their trauma-related stress. A large-scale online relaxation training program is also feasible to help the general public manage their trauma-related stress [57,58]. Efforts at the environmental, political, and structural levels, such as timely and accurate information of the pandemic from official sources (eg, local health agencies and the WHO), may also help to reduce individuals’ stress and panic responses to the COVID-19 pandemic. It is imperative that trusted sources are available to provide risk assessments and recommendations for the general public [59]. Last but not least, promoting healthy use of social media among older people may be particularly beneficial for their social and mental well-being. This population has been vulnerable during the COVID-19 pandemic isolation. Community services should be provided to teach older adults how to use new technology. Policy makers should also pay attention to the potential digital inequality and inequity between generations and should improve accessibility of social media for the older generations.

### Limitations and Future Research

This study has several limitations. First, it was cross-sectional in nature. It is plausible that people with the greatest concerns and depressive symptoms may be more likely to seek out media coverage of the event. Longitudinal studies to monitor the trajectories of social media use and psychological responses are warranted. Second, we recruited the participants via landline telephone numbers, and this sampling method might exclude those without landline telephones or those who were not at home during the survey period (eg, younger adults who are more likely to use mobile phones and less likely to have landline telephones). Thus, this sampling method might also have influenced the representativeness of the sample. Third, this study only focused on the use of SNSs that people use to build social networks or social relationships with other people. We did not investigate the content and functions of the SNSs in this study, or those of other types of SNSs, that may cause different psychological responses to the COVID-19 pandemic [5]. Future studies should investigate these domains of social media use to better understand its impacts on mental health during the COVID-19 pandemic. Fourth, this study used self-reported measures. Thus, the results might be subject to social desirability or recall bias. Fifth, we did not look at other mental health variables, such as anxiety, which may play a role in the mechanisms analyzed in this study. Last but not least, we used item 9 of the PHQ-9 to measure suicidal ideation. Future studies need to validate the results using well-validated scales of suicidal ideation, such as the Suicidal Ideation Questionnaire.

### Conclusions

The findings suggest that social media may be a “double-edged sword” for psychosocial well-being during the COVID-19 pandemic and its roles vary across age groups. The mediators identified in this study should be further validated through qualitative inquiry and longitudinal cohort studies and can be addressed by psychological interventions to prevent severe mental health problems.

## Abbreviations

CESD-10: 10-item Center for Epidemiologic Studies Depression Scale
CFI: comparative fit index
COR: conservation of resources
DSM-IV: Diagnostic and Statistical Manual of Mental Disorders, Fourth Edition
NNFI: nonnormed fit index
PHQ-9: 9-item Patient Health Questionnaire
PTSD: posttraumatic stress disorder
PTSD-8: 8-item Posttraumatic Stress Disorder scale
RMSEA: root mean square error of approximation
SEM: structural equation modeling
SNS: social networking site
WHO: World Health Organization

## References

[1] Wong SY, Kwok KO, Chan FK (2020). What can countries learn from Hong Kong's response to the COVID-19 pandemic?. CMAJ.

[2] Quarantine and isolation. Centers for Disease Control and Prevention.

[3] (2020). Coronavirus Research | March 2020 Release 3: Multi-market research.

[4] Snider M (2020). Video games can be a healthy social pastime during coronavirus pandemic. USA Today.

[5] Chao M, Xue D, Liu T, Yang H, Hall BJ (2020). Media use and acute psychological outcomes during COVID-19 outbreak in China. J Anxiety Disord.

[6] Riehm KE, Holingue C, Kalb LG, Bennett D, Kapteyn A, Jiang Q, Veldhuis CB, Johnson RM, Fallin MD, Kreuter F, Stuart EA, Thrul J (2020). Associations between media exposure and mental distress among US adults at the beginning of the COVID-19 pandemic. Am J Prev Med.

[7] Ni MY, Yang L, Leung CMC, Li N, Yao XI, Wang Y, Leung GM, Cowling BJ, Liao Q (2020). Mental health, risk factors, and social media use during the COVID-19 epidemic and cordon sanitaire among the community and health professionals in Wuhan, China: Cross-sectional survey. JMIR Ment Health.

[8] Gao J, Zheng P, Jia Y, Chen H, Mao Y, Chen S, Wang Y, Fu H, Dai J (2020). Mental health problems and social media exposure during COVID-19 outbreak. PLoS One.

[9] Iob E, Steptoe A, Fancourt D (2020). Abuse, self-harm and suicidal ideation in the UK during the COVID-19 pandemic. Br J Psychiatry.

[10] Pittman M, Reich B (2016). Social media and loneliness: Why an Instagram picture may be worth more than a thousand Twitter words. Comput Human Behav.

[11] Halston A, Iwamoto D, Junker M, Chun H (2019). Social media and loneliness. Int J Psychol Stud.

[12] Sohn Y, Woo S, Jo D, Yang E (2018). The role of the quality of college‐based relationship on social media in college‐to‐work transition of Korean college students: The longitudinal examination of intimacy on social media, social capital, and loneliness. Jpn Psychol Res.

[13] Shah SGS, Nogueras D, van Woerden HC, Kiparoglou V (2020). The COVID-19 pandemic: A pandemic of lockdown loneliness and the role of digital technology. J Med Internet Res.

[14] Liu BF, Kim S (2011). How organizations framed the 2009 H1N1 pandemic via social and traditional media: Implications for US health communicators. Public Relat Rev.

[15] Zhou X, Snoswell CL, Harding LE, Bambling M, Edirippulige S, Bai X, Smith AC (2020). The role of telehealth in reducing the mental health burden from COVID-19. Telemed J E Health.

[16] Depoux A, Martin S, Karafillakis E, Preet R, Wilder-Smith A, Larson H (2020). The pandemic of social media panic travels faster than the COVID-19 outbreak. J Travel Med.

[17] Kramer ADI, Guillory JE, Hancock JT (2014). Experimental evidence of massive-scale emotional contagion through social networks. Proc Natl Acad Sci U S A.

[18] Thompson RR, Jones NM, Holman EA, Silver RC (2019). Media exposure to mass violence events can fuel a cycle of distress. Sci Adv.

[19] Ahmad AR, Murad HR (2020). The impact of social media on panic during the COVID-19 pandemic in Iraqi Kurdistan: Online questionnaire study. J Med Internet Res.

[20] Hobfoll SE (1989). Conservation of resources: A new attempt at conceptualizing stress. Am Psychol.

[21] Feldman DB, Davidson OB, Margalit M (2014). Personal resources, hope, and achievement among college students: The conservation of resources perspective. J Happiness Stud.

[22] van der Velden PG, Setti I, van der Meulen E, Das M (2019). Does social networking sites use predict mental health and sleep problems when prior problems and loneliness are taken into account? A population-based prospective study. Comput Human Behav.

[23] Zhang D, Wang R, Zhao X, Zhang J, Jia J, Su Y, Wang K (2020). Role of resilience and social support in the relationship between loneliness and suicidal ideation among Chinese nursing home residents. Aging Ment Health.

[24] Teo AR, Marsh HE, Forsberg CW, Nicolaidis C, Chen JI, Newsom J, Saha S, Dobscha SK (2018). Loneliness is closely associated with depression outcomes and suicidal ideation among military veterans in primary care. J Affect Disord.

[25] (2019). Social media usage in Hong Kong. Hong Kong Legislative Council Secretariat.

[26] Leist AK (2013). Social media use of older adults: A mini-review. Gerontology.

[27] Berryman C, Ferguson CJ, Negy C (2018). Social media use and mental health among young adults. Psychiatr Q.

[28] Perrin A (2015). Social media usage: 2005-2015. Pew Research Center.

[29] Cellini N, Canale N, Mioni G, Costa S (2020). Changes in sleep pattern, sense of time and digital media use during COVID-19 lockdown in Italy. J Sleep Res.

[30] Sun Y, Li Y, Bao Y, Meng S, Sun Y, Schumann G, Kosten T, Strang J, Lu L, Shi J (2020). Brief report: Increased addictive internet and substance use behavior during the COVID-19 pandemic in China. Am J Addict.

[31] Hansen M, Andersen TE, Armour C, Elklit A, Palic S, Mackrill T (2010). PTSD-8: A short PTSD inventory. Clin Pract Epidemiol Ment Health.

[32] Leung GTY, de Jong Gierveld J, Lam LCW (2008). Validation of the Chinese translation of the 6-item De Jong Gierveld Loneliness Scale in elderly Chinese. Int Psychogeriatr.

[33] Amtmann D, Kim J, Chung H, Bamer AM, Askew RL, Wu S, Cook KF, Johnson KL (2014). Comparing CESD-10, PHQ-9, and PROMIS depression instruments in individuals with multiple sclerosis. Rehabil Psychol.

[34] Campo Arias A, Díaz Martínez LA, Rueda Jaimes GE, Cadena Afanador LDP, Hernández NL (2007). Psychometric properties of the CES-D scale among Colombian adults from the general population. Rev Colomb Psiquiatr.

[35] Björgvinsson T, Kertz SJ, Bigda-Peyton JS, McCoy KL, Aderka IM (2013). Psychometric properties of the CES-D-10 in a psychiatric sample. Assessment.

[36] Cheung C, Bagley C (1998). Validating an American scale in Hong Kong: The Center for Epidemiological Studies Depression Scale (CES-D). J Psychol.

[37] Kroenke K, Spitzer RL (2002). The PHQ-9: A new depression diagnostic and severity measure. Psychiatr Ann.

[38] Tsai F, Huang Y, Liu H, Huang K, Huang Y, Liu S (2014). Patient health questionnaire for school-based depression screening among Chinese adolescents. Pediatrics.

[39] Youth IDEAS (2018). Encouraging the Employment of Seniors.

[40] Lee S, Guo W, Tsang A, Mak AD, Wu J, Ng KL, Kwok K (2010). Evidence for the 2008 economic crisis exacerbating depression in Hong Kong. J Affect Disord.

[41] Qiu J, Shen B, Zhao M, Wang Z, Xie B, Xu Y (2020). A nationwide survey of psychological distress among Chinese people in the COVID-19 epidemic: Implications and policy recommendations. Gen Psychiatr.

[42] Cheng C, Huang J, Liang B (2014). Psychological health diathesis assessment system: A nationwide survey of resilient trait scale for Chinese adults. Stud Psychol Behav.

[43] Roberts T, Miguel Esponda G, Krupchanka D, Shidhaye R, Patel V, Rathod S (2018). Factors associated with health service utilisation for common mental disorders: A systematic review. BMC Psychiatry.

[44] Kramer ADI, Guillory JE, Hancock JT (2014). Experimental evidence of massive-scale emotional contagion through social networks. Proc Natl Acad Sci U S A.

[45] Hobfoll SE (2002). Social and psychological resources and adaptation. Rev Gen Psychol.

[46] Holman EA, Garfin DR, Silver RC (2014). Media's role in broadcasting acute stress following the Boston Marathon bombings. Proc Natl Acad Sci U S A.

[47] Fung IC, Tse ZTH, Cheung C, Miu AS, Fu K (2014). Ebola and the social media. Lancet.

[48] Pahayahay A, Khalili-Mahani N (2020). What media helps, what media hurts: A mixed methods survey study of coping with COVID-19 using the media repertoire framework and the appraisal theory of stress. J Med Internet Res.

[49] Thakur V, Jain A (2020). COVID 2019-suicides: A global psychological pandemic. Brain Behav Immun.

[50] Orben A, Tomova L, Blakemore S (2020). The effects of social deprivation on adolescent development and mental health. Lancet Child Adolesc Health.

[51] Cotten SR, Ford G, Ford S, Hale TM (2014). Internet use and depression among retired older adults in the United States: A longitudinal analysis. J Gerontol B Psychol Sci Soc Sci.

[52] van Ingen E, Rains SA, Wright KB (2017). Does social network site use buffer against well-being loss when older adults face reduced functional ability?. Comput Human Behav.

[53] Baltes PB, Baltes MM, Baltes PB, Baltes MM (1990). Psychological perspectives on successful aging: The model of selective optimization with compensation. Successful Aging: Perspectives from the Behavioral Sciences.

[54] Freund AM, Baltes PB (1998). Selection, optimization, and compensation as strategies of life management: Correlations with subjective indicators of successful aging. Psychol Aging.

[55] Fang Y, Chau AKC, Wong A, Fung HH, Woo J (2018). Information and communicative technology use enhances psychological well-being of older adults: The roles of age, social connectedness, and frailty status. Aging Ment Health.

[56] Vansteenkiste M, Ryan RM, Soenens B (2020). Basic psychological need theory: Advancements, critical themes, and future directions. Motiv Emot.

[57] Ahtinen A, Mattila E, Välkkynen P, Kaipainen K, Vanhala T, Ermes M, Sairanen E, Myllymäki T, Lappalainen R (2013). Mobile mental wellness training for stress management: Feasibility and design implications based on a one-month field study. JMIR Mhealth Uhealth.

[58] Brown JSL, Cochrane R, Hancox T (2000). Large-scale health promotion stress workshops for the general public: A controlled evaluation. Behav Cogn Psychother.

[59] Lachlan KA, Spence PR, Lin X, Najarian K, Del Greco M (2016). Social media and crisis management: CERC, search strategies, and Twitter content. Comput Human Behav.

